# Introduction to Botany of the Marquesas Islands: new taxa, combinations, and revisions

**DOI:** 10.3897/phytokeys.4.1781

**Published:** 2011-07-12

**Authors:** David H. Lorence, Warren L. Wagner

**Affiliations:** 1 National Tropical Botanical Garden, 3530 Papalina Road, Kalaheo, HI 96741 USA; 2 Department of Botany, MRC-166, National Museum of Natural History, Smithsonian Institution, P.O. Box 37012, Washington, DC 20013-7012

The Marquesas Islands (French Polynesia) are an isolated group of volcanic hot spot islands in the SE Pacific Ocean. These 12 islands range from 61.3 to 330 km2 in size, from 360 to 1250 m in elevation, and from 1.3 to 6.3 Ma in age. Steep and rugged, the Marquesas were, until recently, comparatively unexplored and under-collected botanically. The only existing flora for the region was that of (Brown and Brown (1931), Brown 1935 which is incomplete and outdated. Jacques Florence (IRD, Paris) has published two volumes of the Flore de la Polynesie française (Florence 1997, 2004), but this awaits completion. Disturbance by humans, feral animals, and invasive alien plants have severely impacted the lowland and mid-elevation vegetation (Florence and Lorence 1997; Wagner and Lorence 1997).

The currently known native vascular flora comprises about 362 species, of which 45% are endemic. This includes an impressive 118 species of pteridophytes, or 30% of the total. Largest angiosperm lineages are *Psychotria* L., 13 spp., *Bidens* L., 9 spp., *Cyrtandra* J. R. Forst. & G. Forst., 10 spp., *Ixora* L., 7 spp., *Coprosma* J. R. Forst. & G. Forst., 6 spp., *Oparanthus* Sherff,5 spp., and *Kadua* Cham. & Schltdl., 4 spp. Marquesan floristic affinities are with the Society, Austral, and other islands in SE Polynesia, the paleotropics, and to a lesser degree, the Hawaiian Archipelago and the neotropics.

The Vascular Flora of the Marquesas Islands is a collaborative project between the National Tropical Botanical Garden, the Smithsonian Institution, and the Délégation à la Recherche (French Polynesia). In 1987–1988 both of us were taking new research positions at the National Tropical Botanical Garden and the Smithsonian Institution. Our desire to collaborate and to develop interactions between our institutions led to consideration of possible projects. A logical project was a Marquesas flora project because of the years of previous work by Smithsonian researchers Ray Fosberg and Marie-Hélène Sachet in the Marquesas (summarized in Wagner and Lorence 1997), and the focus on Pacific archipelagoes by the National Tropical Botanical Garden. The project was initiated informally on Kauai in 1988 over drinks during a meeting between Peter Raven, who received the Allerton Medal that year, and us. Peter suggested it would be an excellent collaborative project between the two institutions. So with a toast and handshakes the project was born.

The first collaborative field trip in 1988 included staff from the NTBG, Bernice P. Bishop Museum, the Smithsonian Institution, and Jacques Florence of the French research organization ORSTOM. The private yacht of Honolulu resident Ed Carus provided transportation between the islands and served as a mobile field station. Our small team of botanists and entomologists visited the islands of Eiao, Nuku Hiva, Hiva Oa, and Fatu Hiva and made c. 600 collections consisting of more than 2000 specimens, including 10 new species and island records. Two subsequent field trips were funded by NTBG and the Smithsonian, with collaboration from the French Polynesian Delegation for the Environment and ORSTOM. These early trips targeted poorly explored islands and regions on islands. They yielded large numbers of general collections of native and introduced vascular plant species, included a significant number of species new to science in relation to the flora’s relatively small size. The project was continued with new funding by a generous grant from one of the NTBG’s trustees in 2002. This grant enabled us to hire a research technician and conduct field trips to explore and document the flora of many additional islands and habitats. The four collecting expeditions in 2003–2005 yielded c. 6100 herbarium specimens comprising some 714 native and non-native vascular plant species, including additional new, undescribed species and island records. Sixty-two new species were discovered during the course of the project (since 1988), including a significant number by Jacques Florence, increasing the known flora by an impressive 20%.

Based on specimens from these trips several precursor papers in the form of regional taxonomic revisions were published describing new species in the genera *Hedyotis* L. (now included in *Kadua* Cham. & Schltdl.) (Florence & Lorence 2000), *Lepinia* Decne. (Lorence & Wagner 1997), *Trimenia* Seem. (Wagner & Lorence 1999), and *Wikstroemia* Spreng.(Wagner & Lorence 1998). Additional papers describing the vegetation and new species were described in a series of papers published in a special issue of Allertonia documenting the results of the 1988 Fatu Hiva expedition (Lorence 1997). Additional precursor papers from trips in 1995 and 1997 described new species of *Psychotria* L. (Lorence & Wagner 2005), *Ixora* (Lorence & Wagner 2007), and *Elaphoglossum* Schott ex J. Sm. (Rouhan et al. 2008). Additional papers revising the genera *Bidens* (Asteraceae) and *Cyrtandra* (Gesneriaceae) are in preparation.

This series of nine precursor papers in this special issue of PhytoKeys, presented in phylogenetic order, includes three new combinations and descriptions of an additional 18 new species and one new variety of ferns and flowering plants, bringing the total number of new species described from the Marquesas in conjunction with this project and the Flore de la Polynesie francaise to 62. Field work has revealed the majority of these new species to be extremely rare and localized. Consequently, most have been assigned preliminary IUCN Red List ratings of Endangered or Critically Endangered. As has been clearly demonstrated by this project, biodiversity of many tropical islands is still poorly documented and understood. Field work and biological inventories are essential to enhance our knowledge of insular biodiversity and provide critical information for conservation of these organisms and habitats. Results of this project are available on an Internet-based resource hosted on the Smithsonian Department of Botany website [http://botany.si.edu/pacificislandbiodiversity/marquesasflora/index.htm], which provides access to a database of specimens, images, checklist, species pages, elevational range, geographic distribution, and literature. The final goal of this project is publication of a two volume book, the Vascular Flora of the Marquesas Islands.
